# Vegetation and Soil Surface Arthropods in Mediterranean Vineyards with Different Interrow Cover Crop Combinations

**DOI:** 10.3390/insects17090975

**Published:** 2026-09-21

**Authors:** João Roque, Maria Ferreira, Cristina Carlos, Irene Oliveira, António Crespí, Fátima Gonçalves

**Affiliations:** 1 Centre for the Research and Technology of Agro-Environmental and Biological Sciences (CITAB), University of Trás-os-Montes e Alto Douro (UTAD), 5000-801 Vila Real, Portugal; jtroque@utad.pt (J.R.);; 2 Agronomy Department, University of Trás-os-Montes e Alto Douro (UTAD), 5000-801 Vila Real, Portugal; 3 Center for Computational and Stochastic Mathematics (CEMAT), University of Lisbon, 1049-001 Lisbon, Portugal; 4 Department of Mathematics, University of Trás-os-Montes e Alto Douro (UTAD), 5000-801 Vila Real, Portugal

**Keywords:** spontaneous vegetation, sown vegetation, Douro region, activity density, diversity, stability

## Abstract

Interrow cover crops have been increasingly adopted in Mediterranean vineyards to improve soil conservation and other ecosystem services. However, their long-term effects on vegetation dynamics and soil surface arthropod communities remain poorly understood. A study was conducted over three sampling years (2017, 2018 and 2023) in the Douro Demarcated Region (northeastern Portugal) to evaluate, one, two, and seven years after establishment, differences associated with two sown cover crop mixtures, namely a sown legume mixture (LEG) and a sown mixture of legumes and grasses (LEGRA) and spontaneous vegetation (SPONT) in vegetation dynamics and the activity density, richness, diversity, and stability of soil surface arthropod communities. Vegetation was assessed through floristic surveys, and arthropods were sampled using pitfall traps. Vegetation composition and soil surface arthropods community structure varied among sampling years, indicating that temporal variation also contributed to differences in both communities. LEG consistently maintained the highest vegetation cover, whereas LEGRA showed the lowest plant richness. Despite species turnover, LEG and LEGRA retained their functional identity, while SPONT exhibited greater temporal variation in floristic composition. Cover crop treatments were associated with differences in soil surface arthropod activity density, diversity, and community stability. LEGRA showed the highest activity density, whereas higher diversity and evenness were observed in LEG. SPONT presented intermediate values, differing significantly from LEGRA only in Shannon Wiener diversity index. All treatments maintained a relatively balanced trophic structure, suggesting a potential for natural regulation of herbivore population. Vegetation cover and plant richness were positively associated with soil surface arthropod diversity, highlighting the importance of vegetation characteristics for that communities in Mediterranean vineyards.

## Simple Summary

Interrow cover crops have been increasingly adopted in Mediterranean vineyards to mitigate soil erosion and improve biodiversity and soil fertility, but their long-term effects on plant communities and soil surface arthropods remain poorly understood. To address this knowledge gap, we conducted a study over three sampling years (2017, 2018 and 2023) in a vineyard located in the Douro Demarcated Region, in which we compared two sown cover crop mixtures, namely a sown legume mixture (LEG) and a sown mixture of legumes and grasses (LEGRA) with spontaneous vegetation (SPONT). Overall, our findings indicate that cover crop treatments were associated with differences in vegetation and soil surface arthropod communities in vineyards. Vegetation composition and soil surface arthropod community structure varied among sampling years, indicating that temporal variation also contributed to differences in both communities. While the LEGRA mixture showed the highest arthropod activity density, higher diversity and evenness were observed in LEG whereas SPONT presented intermediate values, differing significantly from LEGRA only in Shannon Wiener diversity index. Trophic stability indices indicated relatively balanced trophic structures across all treatments. These results highlight the importance of integrating complementary biodiversity metrics beyond activity density alone and suggest that well-managed spontaneous vegetation may represent a low-input strategy for supporting diverse and functionally balanced soil surface arthropod communities in Mediterranean vineyards.

## 1. Introduction

Soil is the basis of vineyard ecosystems, acting as the primary reservoir of nutrients, organic matter, and water, serving as the habitat for a vast number of organisms that regulate the biogeochemical cycles and directly influence productivity [1,2]. Vineyards represent a major global agricultural system spanning 7.1 million ha and diverse conditions [3], which directly depend on belowground soil health for productivity and ecosystem regulation [4].

At the global scale, vineyards are often managed intensively to optimize grape and wine production, often without considering possible negative impacts on biodiversity and the provision of ecosystem services [5]. Particularly in the Mediterranean region, vineyards are considered among the agricultural systems with the highest soil losses, owing to the combination of steep slopes, intense rainfall events and intensive soil management [6], ultimately compromising soil health and the delivery of essential ecosystem services.

Previous research has shown that plant cover and land use are the main factors controlling soil erosion, often having a greater influence than rainfall intensity or slope gradient [7]. In Mediterranean vineyards, where the soil remains sparsely covered for much of the year, erosion is a major environmental concern because autumn and spring rainfall events can rapidly remove soil, organic matter and nutrients. Consequently, the adoption of soil conservation practices, particularly the replacement of conventional tillage with interrow cover crops, has been recognized as one of the most effective approaches for reducing soil loss and promoting sustainable vineyard management [7].

For many years, vegetation in vineyard interrow was removed due to perceived competition for water and nutrients, and soil was maintained bare through tillage or herbicide applications [8]. However, these practices can negatively affect the soil’s physical, chemical and biological properties, either directly or indirectly, thereby impairing its multifunctionality [8,9]. This management practice, particularly when exacerbated by extreme climate events like intense rainfall occurring over short periods, accelerates soil erosion, thereby degrading soil health and fertility via nutrient and organic matter depletion while driving up atmospheric CO_2_ emissions [9,10,11,12,13]. Consequently, to enhance its sustainability, vineyards should be managed as multifunctional agroecosystems capable of simultaneously maintaining crop productivity, conserving natural resources (soil, water, biodiversity) and enhancing ecosystem services. In response to the growing awareness of this required transition, growers are progressively adapting their soil management practices, shifting toward practices like cover cropping and mulching, although their benefits may be limited in warm, dry regions due to increased competition for water and nutrients.

Four main soil management techniques are commonly used in vineyards: herbicide application, tillage, cover cropping, and mulching. It is rare for a single technique to be applied across the entire vineyard; growers usually combine different techniques for the interrow and row areas. A classic example is the use of cover crops between the rows and cleaning under the row through tillage or herbicide application [12].

Permanent or temporary cover crops has emerged as a sustainable alternative to conventional tillage or herbicide application and has gained increasing adoption across many wine growing regions. As a nature-based solution, cover crops reduce soil erosion, improve soil structure, increase water infiltration and enhance carbon sequestration while simultaneously supporting biodiversity and support multiple ecosystem services, thereby promoting agronomic resilience and contributing to the long-term sustainability of the agricultural systems [8,10,13,14,15,16]. By increasing plant diversity and the input of organic residues, they enhance habitat complexity, regulate soil temperature and moisture, and provide food resources (forage resources, pollen and nectar, residues), overwintering sites and reproduction shelters and refuges for a wide range of organisms. These changes favor a wide range of above and belowground organisms, including microorganisms, pollinators and natural enemies, thereby strengthening ecosystem functioning and contributing to the long-term provision of ecosystem services [13,15]. Moreover, cover crops enhance the ecological and aesthetic value of vineyard landscape, which is particularly relevant in wine-producing regions where viticulture and tourism are closely linked [10].

Soil biota plays a critical role in delivering a wide range of ecosystem services that are essential for the sustainable functioning of natural and managed ecosystems [17,18]. Among soil organisms, arthropods are one of the most diverse and abundant groups, representing up to 85% of soil fauna species richness [19]. Their feeding activity and interactions within the soil food web underpin a wide range of ecological functions, making them key contributors to multiple ecosystem services [20]. Soil arthropods promote decomposition and humification of organic matter, maintenance and fertility, and improve soil structure through soil mixing, pore and void formation, and aggregate formation. By modifying soil physical conditions, hydrology, and the distribution of mineral and organic matter, soil arthropods directly and indirectly regulate the availability of resources for other species. In addition, they provide important services such as pest regulation and seed dispersal [20,21,22,23,24]. Therefore, preserving and enhancing their abundance and diversity is of critical interest to the development of strategies that promote sustainable agriculture [24,25,26].

In addition to their ecological functions, soil arthropods are highly responsive to a wide range of environmental and ecological factors, including habitat disturbance, pollution, and agricultural management practices. This sensitivity has led to their widespread use as bioindicators in agroecosystem assessments [22,27], particularly as indicators of soil quality capable of reflecting broader ecological changes [22,28]. Consequently, arthropod communities have been increasingly incorporated into ecological assessments to evaluate the sustainability of agricultural management practices.

Several studies have shown the positive effect of cover crops on the abundance and diversity of both soil surface and canopy arthropods in vineyards [22,29,30,31,32]. However, the magnitude and direction of these responses depend on the botanical composition, persistence and management of the vegetation cover, as different plant communities provide distinct resources and habitat conditions for arthropods. Recent studies have also shown that strategies of vegetation control influences plant species richness, community composition and functional traits in vineyards [33,34]. Nevertheless, little is known about how the introduction of sown cover crop mixtures influences the long-term dynamics of vineyard vegetation, including species persistence, changes in floristic composition, and the implications of these changes for soil surface arthropod communities.

This study, conducted in a commercial vineyard located in the Douro Demarcated Region, aimed to evaluate the long-term effects of three cover crops treatments—spontaneous vegetation (SPONT), a mixture of sown Fabaceae (LEG), and a mixture of sown Fabaceae and Poaceae (LEGRA)—on vegetation dynamics and on the activity density, diversity, and stability of soil surface arthropod communities. We hypothesized that the establishment of sown cover crop mixtures would alter the long-term dynamics and floristic composition of vineyard vegetation compared with spontaneous vegetation. We further hypothesized that these differences in vegetation composition would promote more abundant and diverse soil surface arthropod communities by increasing habitat complexity and resource availability and would ultimately influence the stability of soil surface arthropod communities. By evaluating the long-term effects of cover crop management on vegetation dynamics and soil surface arthropod communities, this study provides a scientific basis for developing more sustainable vineyard management practices under Mediterranean conditions.

## 2. Materials and Methods

### 2.1. Site Description

The study took place in a commercial vineyard located at Quinta do Casal da Granja, Alijó (41°15′25.59″ N, 7°28′41.13″ W), in the Cima Corgo sub-region of the Douro Demarcated Region in Portugal (Supplementary Figure S1). The area is characterized by a Mediterranean climate, with a mean annual temperature of 13.6 °C and an annual precipitation of around 914 mm [35]. The vineyard of approximately 1.3 ha was on a plain field with a slope lower than 5%. The vines, *Vitis vinifera* L. (cv. Moscatel Ottonel) grafted onto Kober 5BB rootstock, were planted at a spacing of 2.10 m × 0.9 m and trained on a Royat cordon system.

### 2.2. Field Trial

For the field trial, established in November 2016, three experimental blocks of approximately 0.4 ha each (90 m × 44 m, comprising 21 rows with approximately 2100 plants) were established, and assigned to three cover crop treatments: spontaneous vegetation (SPONT), a sown legume mixture (LEG), and a sown mixture of legumes and grasses (LEGRA). Within each block, five sub-blocks of 378 m^2^ (30 m × 12.6 m, comprising 5 rows with around 200 plants) were established (Supplementary Figure S1). The experimental design, based on large treatment blocks rather than smallest and randomized blocks, was adopted for the long-term maintenance of the treatments and to minimize edge effects and interactions between experimental blocks and adjacent areas [36]. The use of large blocks was also intended to reduce potential interference between treatments resulting from the transfer of seeds between cover crop treatments by wind- or arthropod-mediated dispersal. This consideration was particularly relevant given that soil surface arthropods may exhibit aggregated spatial distributions and differ considerably in their movement capacity among taxa [37,38,39]. In November of 2016, all three blocks were tilled. The LEG treatment was sown with a commercial mixture (REVIN LEG ^®^, Fertiprado, Portugal) composed of six Fabaceae species, namely: *Ornithopus sativus* Brot., *Trifolium incarnatum* L., *T. isthmocarpum* Brot., *T. michelianum* Savi, *T. resupinatum* ssp. *resupinatum* L. and *T. subterraneum* spp. *subterraneum* L. The LEGRA treatment was sown with a commercial mixture (REVIN LEGRA ^®^, Fertiprado, Vaiamonte, Portugal) of six Fabaceae and four Poaceae species, namely: *O. sativus* Brot., *T. incarnatum* L., *T.isthmocarpum* Brot., *T. michelianum* Savi, *T. resupinatum* ssp. *resupinatum* L., *T. subterraneum* ssp. *subterraneum* L., *Dactylis glomerata* L., *Festuca rubra* L., *Lolium multiflorum* Lam. var. *westerwoldicum* Wittm and *L. perenne* L. Both seed mixtures were sowed at a rate of 25 kg per ha. In the SPONT treatment, spontaneous vegetation was allowed to develop naturally. The cover crops of the interrows were mown twice each year, in mid-March and mid-July, while vegetation beneath the vine was controlled chemically. All other vineyard management practices, including phytosanitary treatments, fertilization, and cultural operations, were included in the sown mixtures qual across the three treatments throughout the study period.

### 2.3. Data Sampling

The experiment was monitored during the first two years after its establishment (2017 and 2018) and reassessed seven years later (2023).

#### 2.3.1. Floristic Survey of Cover Crops Vegetation

The floristic surveys were conducted annually, between late May and early June, following the methodology previously described [40]. A fixed 2 m × 2 m sampling grid centered on the pitfall trap located in the interrow was used to include vegetation from both the vine row and the interrow. Depending on the different management treatments applied to these two cultivated zones, the resulting vegetation composition varies, allowing for the assessment of floristic and structural differences, resulting in a total of 10 sampling units per cover crop treatment. For each area, all plant species were identified and recorded, as well as the abundance and cover of each species observed in each sampling area.

#### 2.3.2. Soil Surface Arthropods

Soil surface arthropods were monitored using pitfall traps in 2017 on 20 April and 18 May (spring), 14 July and 25 August (summer), and 25 September (autumn); in 2018 on 11 May (spring), 25 June, 30 July and 7 September (summer) and 8 October (autumn); and in 2023 on 24 April and 15 June (spring) and 31 July (summer). As a passive and non-selective sampling method, pitfall traps capture a wide range of soil surface arthropods over time, allowing for the assessment of temporal variations in their activity [41]. Accordingly, this method was therefore selected to assess the activity density of arthropods active at the soil surface, rather than to estimate the absolute density of the soil arthropod community [42]. This approach was considered appropriate for the objectives of the present study, which focused on the temporal variation in the activity density of the soil surface arthropod community. These traps consist of a container (plastic cups with around 9 cm of height and 7 cm of diameter) buried in the soil with its opening flush with the soil’s surface and partially filled with a preservative solution (25% ethylene glycol solution) containing a few drops of detergent to reduce surface tension), to retain and preserve captured species.

Two traps were installed per sub-block along the central vine interrow, with a distance of 20 m between traps, resulting in a total of 10 traps per cover crop treatment and 30 traps per sampling date. The traps remained in the field for 96 h, after which the trap contents were transferred to labelled flasks and stored in a fridge until observation. The exposure period of 96 h was selected to minimize potential reductions in trapping efficiency and deterioration of captured specimens, particularly under the high temperatures prevailing during the warmer sampling periods in Mediterranean vineyards. Arthropods were initially sorted according to the Rapid Biodiversity Assessment (RBA) [43,44]. Specimens were then counted and identified to the lower taxa level possible (normally family or order) [22]. For Carabidae and most of Formicidae, identification was possible to the genus or species level. Finally, they were also classified according to their trophic group. Thus, following the literature, arthropods were assigned to five categories: detritivores; herbivores; omnivores; parasitoids; and predators [45,46,47,48,49,50,51]. As only a few parasitoids were collected, this group was merged with predators to produce a “zoophages” category. Arthropods whose feeding guild could not be determined were included in an indeterminate group.

### 2.4. Calculation of Response Variables

#### 2.4.1. Cover Crops

Species richness (S) was calculated as the total number of plant species recorded in each sampling unit.

Vegetation cover (%) was calculated as

Cover (%) = (A_t_/A_s_) × 100,

where A_t_ is the area occupied by vegetation and A_s_ is the total sampling area (1 m^2^).

#### 2.4.2. Soil Surface Arthropod Activity Density and Diversity

The activity density, richness, and Simpson’s diversity index (SDI), Shannon–Wiener’s Index (H’) and Pielou’s Index (J) of the soil surface arthropods were calculated as follows:

Activity density = N,

where N represents the total number of individuals.

Richness = S,

where S represents the total number of morphospecies.

Simpson’s Diversity Index (SDI) = SDI = ∑(n/N)^2^,

where n represents the total number of individuals of the same morphospecies and N represents the total number of individuals. This Index represents the probability that two individuals randomly selected from a sample belong to the same morphospecies [52], and ranges from 0 to 1, with values closer to 1 indicating lower diversity.

Shannon–Wiener’s Index (H’) = −∑ pi × ln(pi),

where pi is the proportion of individuals of a morphospecies to the total number of individuals in the community. This index quantifies diversity within a community by integrating both morphospecies richness and its relative abundance. It reaches its maximum value when all species are equally abundant [53].

Pielou’s evenness index (J) = H’/H_max_,

where:

H_max_ = ln(S)

S is the total number of species in the community and H’ is the Shannon–Wiener’s Index. It measures the uniformity of the distribution of individuals among the morphospecies within a community. It ranges from 0 to 1, with values closer to 1 indicating that individuals are more evenly distributed and values close to 0 indicating that one or a few species dominate the community, whereas the remaining species are represented by relatively few individuals [53].

In order to facilitate the interpretation of the diversity results, Hill numbers _q_D were also calculated, as they express diversity as the effective number of species [54,55].

When q = 1, the Hill number ^1^D, corresponds to exponential of the Shannon–Wiener index and represents the effective number of common species, reflecting the equity of the community:

^1^D = e^H’^

When q = 2, the Hill number ^2^D, correspond to the inverse of Simpson’s diversity index and represents the effective number of dominant species, indicating the ecological dominance within the community:

^2^D = 1/SDI

#### 2.4.3. Stability of Soil Surface Arthropod Communities

Based on the soil surface arthropod data, four stability indexes were calculated to assess the balance between herbivorous, zoophagous and neutral arthropods within the soil surface arthropod community [56]. For the calculation of these indices, neutral arthropods comprised omnivores and detritivores [56]. Here, the term “neutral” refers specifically to the trophic classification used in the stability indices, with these arthropods considered neither herbivorous nor zoophagous, and does not imply an absence of ecological function. Neutral arthropods were included because their contribution to arthropod community stability has been previously demonstrated [56]. Two indexes were calculated using the activity density of functional groups:

N_ne_/N_h_

and

(N_ne_ + N_n_)/N_h_,

where N_ne_, N_h_ and N_n_ represent the activity density of zoophagous, herbivorous and neutral arthropods, respectively.

Two additional indices were calculated using the richness of the functional groups:

S_ne_/S_h_

And

(S_ne_ + S_n_)/S_h_,

where S_ne_, S_h_ and S_n_ represent the richness of zoophagous, herbivorous and neutral arthropods, respectively.

Higher values of these indices indicate a greater relative activity density or richness of zoophagous and neutral arthropods in relation to herbivores, suggesting a more stable arthropod community and a greater potential for the natural regulation of herbivore populations.

### 2.5. Statistical Analysis

Analyses were performed with R (version 4.5.1) [57]. The significance level was set at α = 0.05 for all statistical tests.

Indicator species analysis was performed following Dufrêne and Legendre [58] and using the “labdsv” package version: 2.3-1 [59] to identify plant species associated with each treatment. The method combines species specificity (relative abundance within a treatment) and fidelity (frequency of occurrence among samples of that treatment) to calculate an indicator value.

Stratigraphic plots for the percentage cover of each plant species for plant species identified as significant indicator by the IndVal analysis was created using “rioja” package version 1.0-7 [60].

Community composition of plants and soil surface arthropods among treatments and years was analyzed by non-metric multidimensional scaling (NMDS) implemented in the “vegan” package version 2.7-6 [61]. In the case of arthropods, individuals were grouped by functional group and by taxa (normally order level). Only groups with more than 25 individuals (in total) were included in the analyses. Several dissimilarity indices available in the vegdist() function were evaluated, and the final two-dimensional NMDS solutions were selected according to the lowest stress values and ecological interpretability.

Associations between NMDS axes and treatments, or years, were assessed by ANOVA, after checking normality, or Kruskal–Wallis. For arthropods, relationships between the ordination and vegetation cover percentage and species richness were assessed using the envfit() function, using “ggplot2” pakage version 4.0.3 [62].

Generalized linear mixed models (GLMMs) were fitted using the “lme4” pakage version 2.0-6 [63] and the glmer() function to evaluate the effects of treatment, year and, for arthropods, season. Response variables were modelled assuming Poisson distributions with log links for count data (activity density and richness) and Gaussian distributions with identity links for continuous variables (cover percentage, diversity indices and stability metrics). Model assumptions and goodness-of-fit were evaluated, and the most appropriate error distribution was selected for each response variable.

To account for the hierarchical structure of the experimental design and the non-independence of observations within experimental units, random intercepts were included for subplots nested within treatment and, where applicable, for sampling points nested within subplots and treatment.

Accordingly, the general model structure for vegetation responses was

Response ~ Treatment + Year + (1 | Treatment:Subplot) + (1 | Treatment:Subplot:Point)

where Treatment and Year were included as fixed effects, while Subplot and Point were represented by random intercepts reflecting the nested experimental structure. The point-level random effect, (1 | Treatment:Subplot:Point), was included because two sampling points were sampled within each subplot.

For arthropod responses, season was additionally included as a fixed effect:

Response ~ Treatment + Year + Season + (1 | Treatment:Subplot) + (1 | Treatment:Subplot:Point)

Vegetation variables were not included as explanatory variables in the arthropod GLMMs because vegetation was sampled only once per year, whereas arthropods were sampled seasonally. The significance of fixed effects was evaluated using Wald χ^2^ tests, followed by Tukey-adjusted pairwise comparisons. Nested boxplots were generated using the “tidyverse” version 2.00 [64] and “ggh4x” version 0.3.1 [65] packages.

Pearson correlation coefficients were calculated to assess relationships between vegetation attributes (species richness and percentage cover) and arthropod community metrics (activity density, diversity indices and community stability).

## 3. Results

### 3.1. Cover Crop Dynamics Across Treatments and Years

The floristic survey conducted in the vineyard interrow allowed the identification of plant species present and the estimation of the percentage of vegetation cover in each cover crop treatment and sampling year. Across the three study years, a total of 74 plant species were identified (Supplementary Table S1).

As expected, several species included in the sown mixtures (i.e., *Dactylis glomerata*, L. *multiflorum*, *O. sativus*, *T. incarnatum*, *T. isthmocarpus*, *T. resupinatum*, *T. subterraneum*) also occurred spontaneously in the SPONT treatments, indicating that these species were already present in the soil seed bank.

In the LEG treatment, five of the six sown species were recorded in 2017 (Supplementary Table S1). By 2023, only two of these species remained. *O. sativus*, *T. incarnatum* and *T. resupinatum* ssp. *resupinatum* were recorded in 2017 and 2018 but were no longer present in 2023. *T. isthmocarpum* was recorded only in 2017, whereas *T. michelianum* was first recorded in 2018 and remained present until 2023. *T. subterraneum* was recorded throughout the all study period (2017, 2018 and 2023), which indicates greater efficiency of this species in the sown combinations applied. In the LEGRA treatment, eight of the ten sown species were recorded in 2017, whereas only three persisted until 2023. *D. glomerata*, *T. michelianum* and *T. subterraneum* ssp. *subterraneum* were present in all the three sampling years. Although included in the seed mixture, *F. rubra* and *L. perenne* were not recorded in any year. *L. multiflorum var. westerwoldicum*, *O. sativus*, *T. incarnatum* and *T. resupinatum* ssp. *resupinatum* were recorded in 2017 and 2018 but were absent in 2023. *T. isthmocarpum* was recorded only in 2017.

The IndVal analysis identified several significant indicator species associated with the different cover crop treatments in each survey year and in the pooled dataset (Supplementary Table S2). Although some indicator species were consistently detected across years, others were restricted to individual sampling years, suggesting temporal changes in vegetation composition and habitat conditions. The pooled analysis (2017–2018–2023) identified the most consistent indicators, reflecting stable ecological associations throughout the study period.

**Figure 1:**
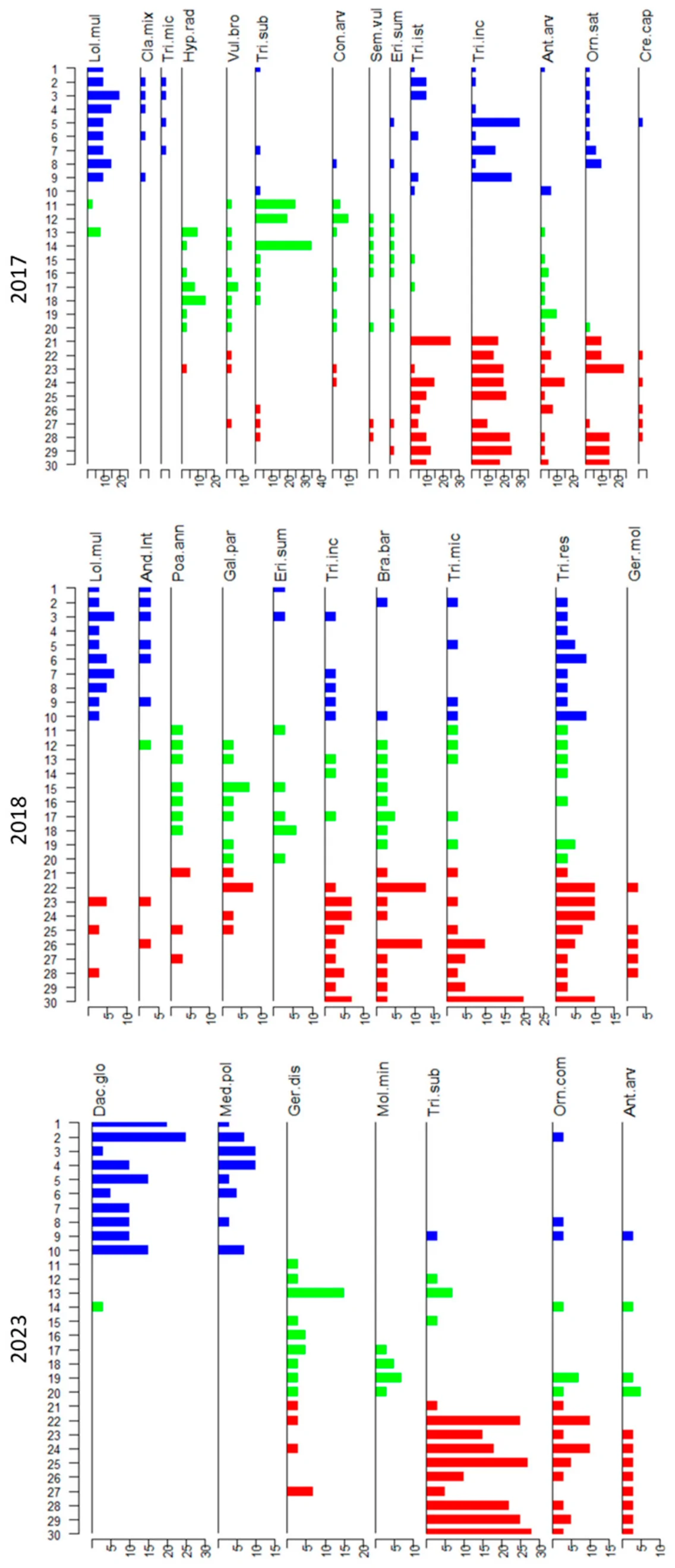
Stratigraphic plot showing the percentage cover of individual plant species with significant indicator values (IndVal, *p* < 0.05) for the 2017, 2018 and 2023 Numbers (1–30) correspond to the sampling points. Colors represent the three treatments: LEGRA (blue), SPONT (green), and LEG (red).

In the sown cover crops, although the identity of the indicator species changed over time, the communities retained the floristic pattern of the original seed mixtures. In LEGRA, *L. multiflorum* was the main indicator species in 2017 and 2018, with high IndVal scores (0.818 and 0.793, respectively). By 2023, however, *D. glomerata* became the characteristic species of this treatment (IndVal = 0.976). In contrast, no persistent indicator species were detected in the SPONT treatment across the three sampling years, with a complete turnover of indicator species over time. In LEG, although the identity of the indicator species also changed, they consistently belonged to the same functional group (Fabaceae). In 2017, LEG was characterized by *T. isthmocarpum* (IndVal = 0.628) and *T. incarnatum* (IndVal = 0.600). The latter remained an indicator species in 2018 (IndVal = 0.578), whereas in 2023, *T. subterraneum* became the main indicator species (IndVal = 0.918). These temporal shifts are illustrated in Figure 1, which shows the stratigraphic distribution and percentage cover of each species with significant indicator values across treatments over the study period.

**Figure 2:**
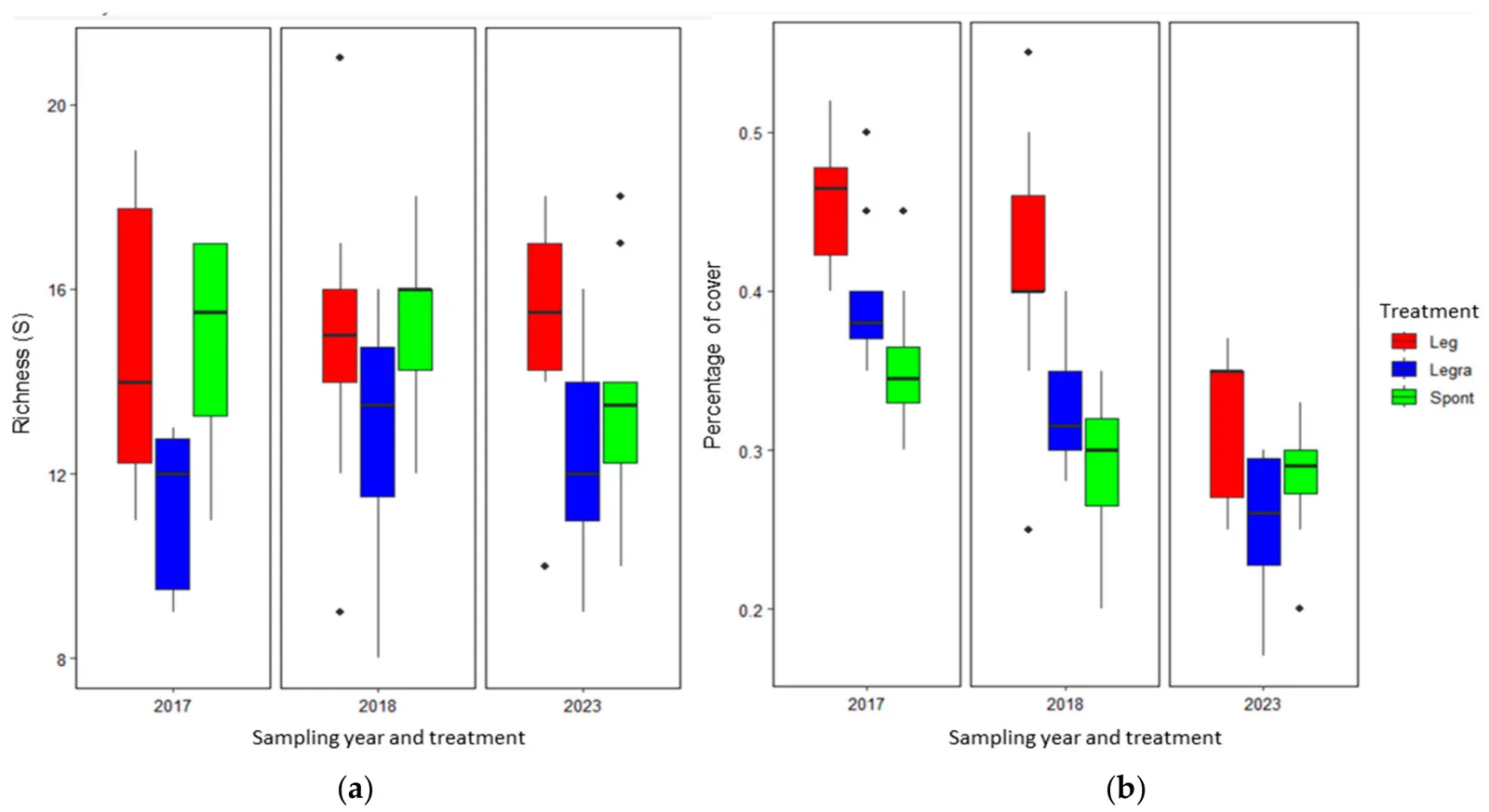
Species richness (**a**) and percentage cover (**b**) of each treatment (mean ± SE).

GLMM analysis revealed significant effects of explained variables on plant richness (Wald χ^2^ = 10.5; df = 4; *p* = 0.033). The lowest values of richness were recorded in LEGRA treatment (11.3 ± 1.7 to 13.1 ± 2.5) than in the LEG (14.8 ± 2.9 to 15.3 ± 2.3) and SPONT treatment (13.6 ± 2.5 to 15.2 ± 2.0) (Wald χ^2^ = 9.8; df = 2; *p* = 0.0076). The later ones did not differ between them (Figure 2a). The year had no significant effect on plant richness (Wald χ^2^ = 0.74; df = 2; *p* = 0.69).

Regarding percentage of cover, the analysis also revealed significant effects of both explained variables on it (Wald χ^2^ = 129.3; df = 4; *p* < 0.001). The highest values were recorded in LEG treatment (31.9 ± 4.7 to 45.5 ± 4.1%) compared to the LEGRA treatment (14.8 ± 2.9 to 39.5 ± 4.7%) and SPONT (28.1 ± 3.6 to 35.2 ± 4.6%) (Wald χ^2^ = 51.1; df = 2; *p* < 0.001). The later ones did not differ between them (Figure 2b). The year also had a significant effect on percentage of cover (Wald χ^2^ = 78.1; df = 2; *p* = *p* < 0.001).

**Figure 3:**
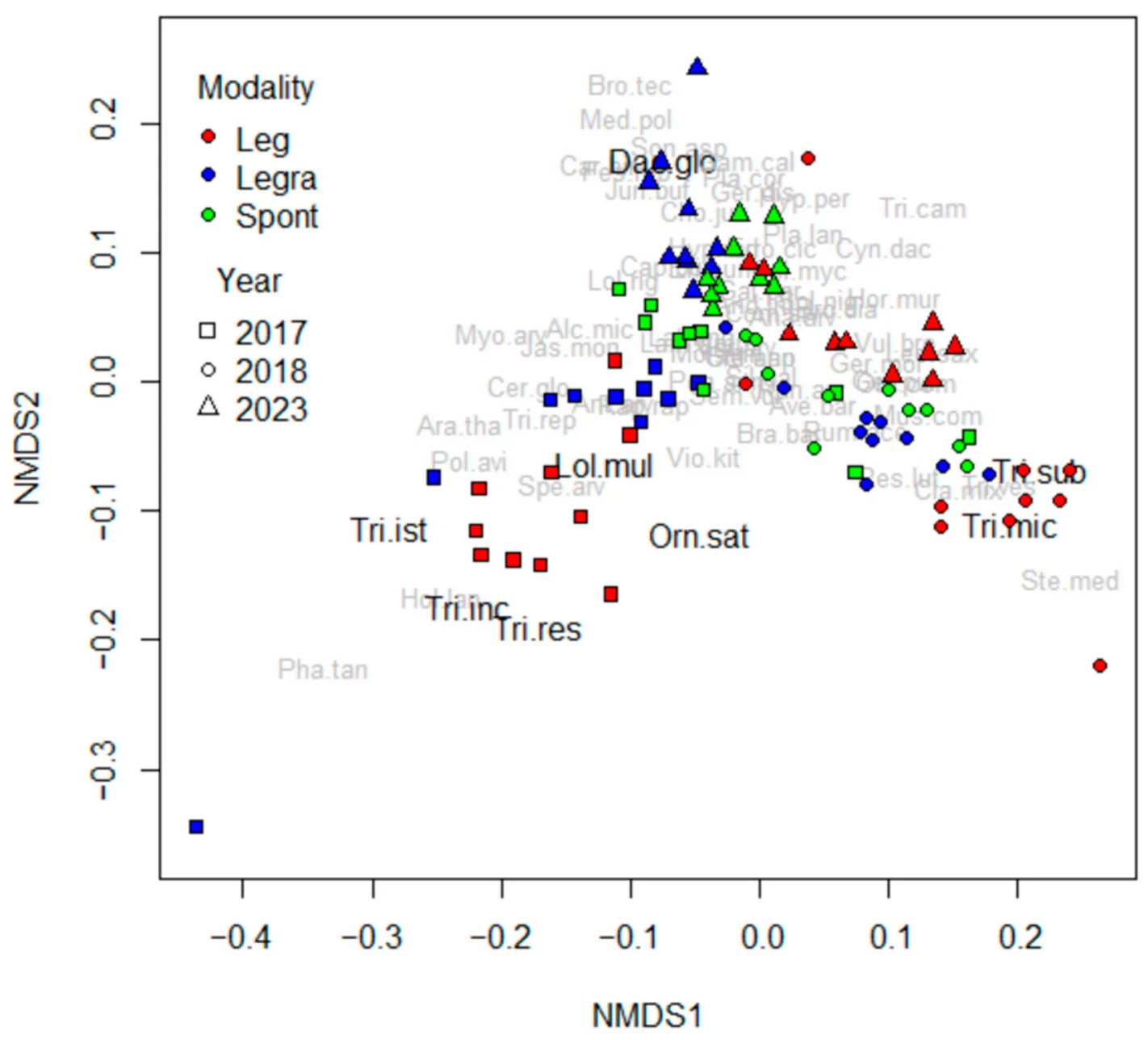
Two-dimensional non-metric multidimensional scaling (NMDS) ordination of plant community composition based on the percentage cover of each plant species using Euclidean dissimilarity. Colors indicate cover crop treatments and symbols represent survey years. Plant species included in the original seed mixtures of the LEG and LEGRA treatments are highlighted in bold and larger font. Grey labels correspond to the remaining species. Stress = 0.134. Abbreviations are defined in Supplementary Table S1.

The final two-dimensional NMDS ordination of percentage cover of each plant species, using Euclidean dissimilarity, produced a stress value of 0.134, indicating a moderate quality (Figure 3). Along NMDS1, significant differences were detected among years (F_(2, 85)_ = 51.883, *p* < 0.001) and treatments (F_(2, 85)_ = 5.474, *p* = 0.006). Similarly, NMDS2 separated years (F_(2, 85)_ = 40.400, *p* < 0.001) and treatments (F_(2, 85)_ = 11.040, *p* < 0.001), indicating that temporal changes in vegetation composition were more pronounced than differences among cover crop treatments. In 2023, communities clearly displaced from those sampled in 2017, while the 2018, communities occupied an intermediate position. Although the separation among treatments was less pronounced, LEG, LEGRA, and SPONT showed distinct floristic patterns, consistent with the indicator species analysis. In particular, LEGRA became increasingly associated with *D. glomerata*, whereas LEG maintained a floristic composition dominated by Fabaceae species throughout the study period.

### 3.2. Overall Composition of the Soil Surface Arthropod Community

During the study, a total of 92,739 soil surface arthropods belonging to 381 morphospecies were collected (Supplementary Table S3). Collembola was the most abundant taxonomic group, accounting for 67.9% of all collected individuals, followed by Hymenoptera (Formicidae) (18.0%), Coleoptera (4.0%), Acari (3.5%), Araneae (3.1%) and Hemiptera (1.60%).

Soil surface arthropods caught were classified into four trophic groups: detritivores (71.3%), herbivores (12.0%), zoophages (5.8%), and omnivores (8.5%). Detritivores included Blattodea, Coleoptera (Aderidae, Anthicidae, Corylophidae, Latridiidae, Scarabaeidae, Tenebrionidae), Collembola, Diplopoda, Isopoda andOribatida. Herbivores included Coleoptera (some Carabidae—*Amara* and *Harpalus* genera—, Chrysomelidae, Curculionidae, Dasytidae, Elateridae, Nitidulidae, Phalacridae), Hemiptera (Aphididae, Cercopidae, Cicadellidae, Cydnidae, Psyllidae, Lygaeidae, Pentatomidae), Hymenoptera (Formicidae, *Messor barbarus*), and Orthoptera (Acrididae, Gryllidae, Gryllotalpidae). Zoophages included Araneae, Coleoptera (Carabidae, Coccinellidae, Malachiidae, Meloidae, Staphylinidae), Chilopoda, Hemiptera (Anthocoridae, Nabidae), Diptera (Syrphidae), Hymenoptera (Chalcidoidea, Chrysididae, Dryinidae, Icheneumonoidea, Mutillidae, Pompilidae, Vespidae), Opiliones and Scorpiones. Omnivores comprised the remaining Formicidae. Arthropods whose trophic guild could not be determined (indeterminate) accounted for 2.4% of all collected individuals and included other Acari, Diptera, Miridae and other Heteroptera, as well as unidentified families of Coleoptera and Thysanoptera.

The relative abundance of detritivores, mainly represented by Collembola, ranged from 56.9% in LEG to 81.1% in LEGRA and increased over time, from 58.1% in 2017 to 75.6% in 2023 (Supplementary Figure S2a,b). The mean activity density of detritivores per pitfall trap ranged from 32.53 ± 10.00 in LEG in 2023 to 861.77 ± 324.24 in LEGRA in the same year. Mean richness ranged from 1.48 ± 0.11 in SPONT to 1.67 ± 0.18 in LEGRA, both in 2023 (Supplementary Table S4). Herbivores, largely represented by the seed-harvesting ant *M. barbarus*, accounted for 7.1% in LEGRA and 21.6% in LEG (Supplementary Figure S2a). Across years, herbivores represented between 10.1% (2018) and 15.8% (2017) (Supplementary Figure S2b). Their mean activity density per pitfall trap ranged from 16.02 ± 2.92 in LEGRA in 2018 to 70.57 ± 25.89 in LEG in 2023. Mean richness ranged from 2.22 ± 0.23 in SPONT in 2018 to 4.27 ± 0.22 in LEG in 2023 (Supplementary Table S4). Omnivores represented between 6.1% in LEGRA and 11.3% in LEG, and between 6.5% in 2018 and 15.4% in 2017 (Supplementary Figure S2a,b). Their mean activity density per pitfall trap ranged from 14.56 ± 1.84 in SPONT in 2018 to 33.63 ± 4.36 in LEG in 2023, while mean richness ranged from 2.93 ± 0.19 in SPONT in 2023 to 4.07 ± 0.26 in SPONT in 2017. Zoophages accounted for between 3.5% in LEGRA and 8.6% in SPONT, whereas across years they ranged from 3.7% in 2023 to 9.3% in 2017 (Supplementary Figure S2a,b). The mean activity density of zoophagous arthropods per pitfall trap ranged from 11.96 ± 1.64 in LEGRA in 2017 to 15.87 ± 4.27 in LEGRA in 2023, while mean richness ranged from 5.02 ± 0.34 in LEG in 2018 to 7.45 ± 0.73 in LEG in 2017 (Supplementary Table S4).

### 3.3. Effect of Cover Crop Treatment, Season, and Year on Soil Surface Arthropod Activity Density and Diversity

The global model revealed significant effects of cover crop treatment, season and sampling year on soil surface arthropod activity density (Wald χ^2^ = 47,655.4; df = 6; *p* < 0.001). Activity density was significantly higher in the LEGRA treatment than in the other treatments that did not differ between them (Wald χ^2^ = 8.1; df = 2; *p* = 0.017). In fact, 52.4% of arthropods were collected in LEGRA, against 25.8% in LEG and 21.8% in SPONT. Arthropod activity density was also significantly higher in spring and lower in autumn (Wald χ^2^ = 35,192.9; df = 2; *p* < 0.001). The three years differed significantly, with activity density being significantly higher in 2023 than in the other treatments; the activity density also was higher in 2018 than in 2017 (Wald χ^2^= 14,199.1; df = 2; *p* < 0.001) (Table 1).

**Figure 4:**
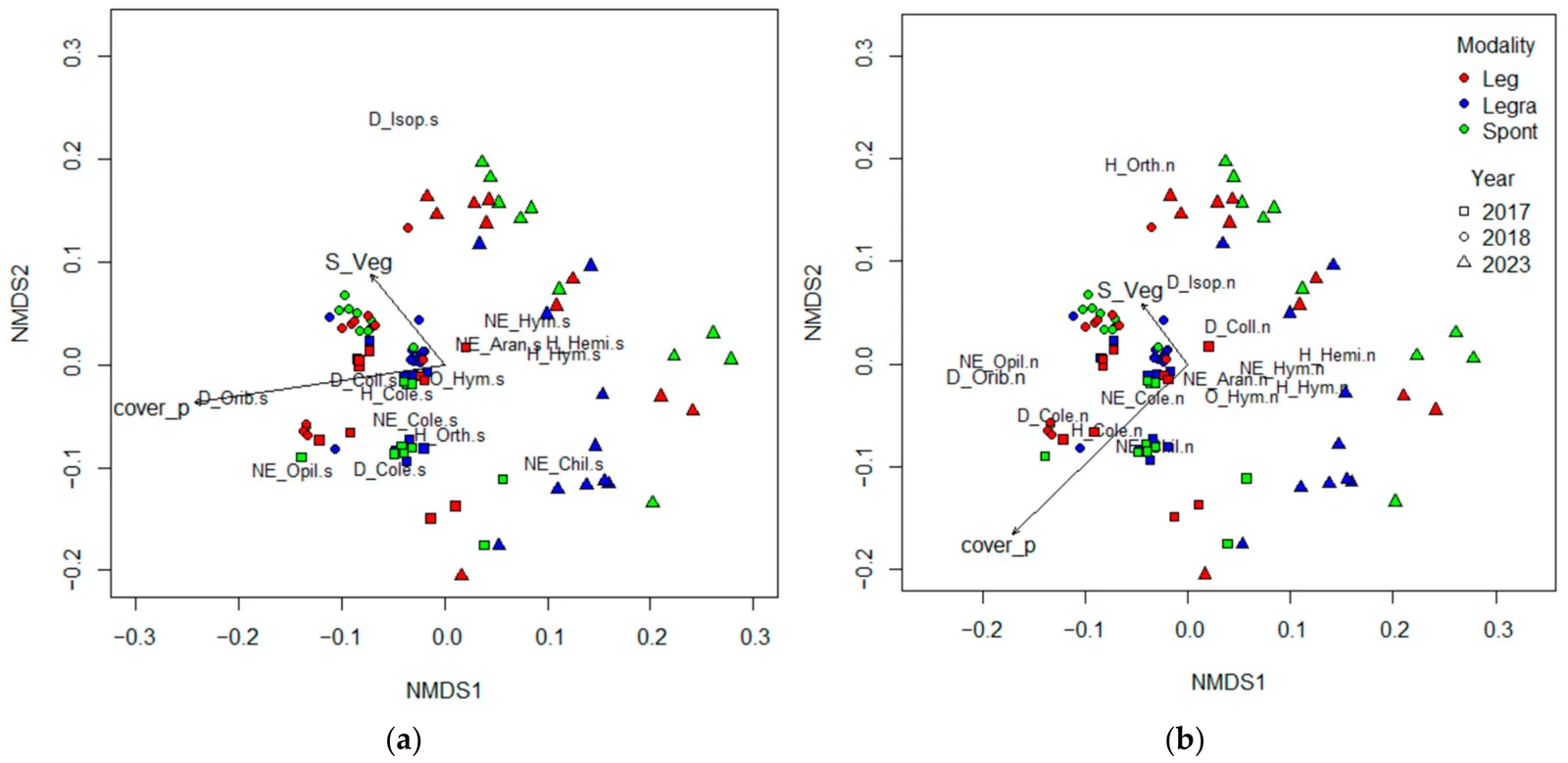
NMDS ordination plots of the richness (**a**) and activity density (**b**) of trophic groups of soil surface arthropods in three analyzed years, in three different cover crop treatments (SPONT: spontaneous vegetation; LEG: sown cover crop with a mixture of Fabaceae species; LEGRA: sown cover crop with a mixture of Fabaceae and Poaceae species). D—detritivore; H—herbivore; NE—natural enemy; O—omnivore; Aran—Araneae; Coll—Collembola; Col—Coleoptera; Hemi—Hemiptera; Hym—Hymenoptera; Isop—Isopoda; Ort—Orthoptera; Opil—Opiliones; Orib—Oribatida. Fitted environmental variables: cover_p—percentage of cover vegetation; S_Veg—plant richness.

The final two-dimensional NMDS solution for arthropod activity density was obtained using the Euclidean dissimilarity measure (stress = 0.19, indicating a low-to-moderate-quality ordination) (Figure 4b). In NMDS1 significant differences were detected among years (χ^2^ = 60.632, df = 2, *p* < 0.001), whereas no differences were detected among treatments (χ^2^ = 0.634, d.f = 2, *p* = 0.728). Pairwise comparisons showed that samples collected in 2023 differed significantly from both 2017 and 2018 (Bonferroni-adjusted *p* < 0.001), while no differences were found between 2017 and 2018. In contrast, in NMDS2 no significant differences were detected among years (F_(2, 85)_ = 1.802, *p* = 0.171) or treatments (F_(2, 85)_ = 2.611, *p* = 0.079). Fitting environmental variables to the ordination revealed that percentage of cover vegetation (cover_p) was significantly associated with the activity density of arthropod assemblage (r^2^ = 0.245, *p* = 0.001), although the correlations with the individual NMDS axes were only moderate (NMDS1 = −0.42; NMDS2 = −0.259). Conversely, plant richness (S_Veg) was not significantly related to the ordination (r^2^ = 0.021, *p* = 0.365).

Soil surface arthropod richness was significantly affected by season and sampling year (Wald χ^2^ = 361.8; df = 6; *p* < 0.001). The highest values were recorded in spring and the lowest in autumn (Wald χ^2^ = 318.0; df = 2; *p* < 0.001). Regarding the sampling year, significant differences were also observed, with the highest richness recorded in 2017 and the lowest in 2018 (Wald χ^2^ = 39.8; d.f = 2; *p* < 0.001). Cover crop treatment had no significant effect on richness (Wald χ^2^ = 1.5; df = 2; *p* = 0.470) (Table 1).

The final two-dimensional NMDS solution for arthropod richness was obtained using the Euclidean dissimilarity measure (stress = 0.199, indicating a low-to-moderate-quality ordination) (Figure 4a). In NMDS1 significant differences were detected among years (F_(2, 85)_ = 30.995, *p* < 0.001), whereas no significant differences were observed among treatments (F_(2, 85)_ = 2.323, *p* = 0.104). Tukey’s post hoc test indicated significant differences among all sampling years, with 2017, 2018 and 2023 forming three distinct groups (*p* < 0.01). In the NMDS2, no significant differences were detected among treatments (Kruskal–Wallis, χ^2^ = 1.154, d.f = 2, *p* = 0.562), whereas significant differences were detected among years (Kruskal–Wallis, χ^2^ = 32.514, d.f = 2, *p* < 0.001), except between 2017 and 2018. Pairwise comparisons showed that samples collected in 2023 differed significantly between all years. Among the fitted environmental variables, vegetation cover percentage (cover_p) was significantly related to the arthropod community ordination (r^2^ = 0.262, *p* = 0.001), although the correlations with the ordination axes were only weak-to-moderate (NMDS1 = 0.371; NMDS2 = − 0.352). In contrast, plant richness (S_Veg) was not significantly associated with arthropod richness ordination (r^2^ = 0.046, *p* = 0.138), presenting only weak correlations with the NMDS axes.

Regarding the Shannon–Wiener diversity index (H’), analysis revealed significant effects of the three explained variables (Wald χ^2^ = 43.8; df = 6; *p* < 0.001). H’ was significantly higher in LEG than in LEGRA treatment, whereas SPONT exhibited intermediate values that did not differ significantly from either treatment (Wald χ^2^ = 13.5; df = 2; *p* = 0.0012). H’ was significantly higher in the spring and summer seasons than in the autumn (Wald χ^2^ = 11.0; df = 2; *p* = 0.0041) and significantly higher in 2017 than in 2018 and 2023 whereas this two last years did not differ (Wald χ^2^ = 21.0; df = 2; *p* < 0.001) (Table 1).

Significant effects of the cover crop treatment, season and year were obtained for the Simpson’s diversity index (SDI) (Wald χ^2^ = 32.5; df = 6; *p* < 0.001). SDI was significantly higher in the LEGRA treatment than in the LEG and SPONT treatments (Wald χ^2^ = 14.5; df = 2; *p* < 0.001); SDI was significantly higher in the spring than in the summer, while autumn showed intermediate values, with no significant differences from either season (Wald χ^2^ = 10.2; df = 2; *p* = 0.006). SDI was also significantly lower in 2017 than in 2018 and 2023, whereas no significant differences were detected between the latter two years (Wald χ^2^ = 13.6; df = 2; *p* = 0.0011) (Table 1).

Also, Pielou’s evenness index (J) was significantly affected by the three explained variables (Wald χ^2^ = 46.3; df = 6; *p* < 0.001). J was significantly higher in LEG and SPONT treatments than in the LEGRA treatment (Wald χ^2^ = 13.5; df = 2; *p* = 0. 0012), significantly higher in summer and autumn than in spring (Wald χ^2^ = 24.0; df = 2; *p* < 0.001), and significantly higher in 2017 and 2023 than in 2018 (Wald χ^2^ = 14.9; df = 2; *p* 0.006) (Table 1).

Regarding Hill numbers, both ^1^D and ^2^D were significantly affected by the three explained variables (Wald χ^2^ = 75.1; df = 6; *p* < 0.001, for ^1^D and Wald χ^2^ = 41.7; df = 6; *p* < 0.001 for ^2^D). ^1^D was significantly higher in LEG treatments than in LEGRA treatment while SPONT presented intermediate values (Wald χ^2^ = 10.3; df = 2; *p* < 0.006). It was also significantly higher in spring than in summer and autumn, and significantly higher in summer than in autumn (Wald χ^2^ = 29.1; df = 2; *p* < 0.001). Regarding the sampling year, ^1^D was significantly higher in 2017 than in 2018 and 2023 (Wald χ^2^ = 35.7; df = 2; *p* < 0.001). Also, ^2^D was significantly higher in the LEG than in the LEGRA treatment while SPONT presented intermediate values (Wald χ^2^ = 9.7; df = 2; *p* = 0.008). It was significantly higher in spring than in autumn, whereas summer did not differ significantly from either spring or autumn (Wald χ^2^ = 8.3; df = 2; *p* 0.016). ^2^D was also higher in 2017 than in 2018 and 2023 (Wald χ^2^ = 22.6; df = 2; *p* < 0.001) (Table 1).

### 3.4. Effect of Cover Crop Treatment, Season and Year on the Stability of the Soil Surface Arthropod Communities

The global model showed significant effects of the season and sampling year on the stability index N_ne_/N_h_ (Wald χ^2^ = 39.7; df = 6; *p* < 0.001). This index was significantly higher in the spring and autumn seasons than in summer (Wald χ^2^ = 16.1; df = 2; *p* = 0.0032) and significantly higher in 2018 than in 2017 and 2023 (Wald χ^2^ = 23.3; df = 2; *p* < 0.001). In contrast, cover crop treatments had no significant effect on this index (Wald χ^2^ = 1.2; df = 2; *p* = 0.55) (Table 2).

Regarding the index (N_ne_ + N_n_)/N_h_ it was observed significant effects of the three explained variables (Wald χ^2^ = 20.1; df = 6; *p* = 0.003). This index was significantly higher in the SPONT treatment than in the LEG treatment, whereas neither treatment differed significantly from the LEGRA treatment (Wald χ^2^ = 7.3; df = 2; *p* = 0.026). It was also significantly higher in spring than in the summer season, while values in autumn dis not differ from other seasons (Wald χ^2^ = 6.5; df = 2; *p* = 0.039) and significantly higher in 2018 than in 2023 whereas 2017 exhibited intermediate values that did not differ significantly from either years (Wald χ^2^ = 10.9; df = 2; *p* = 0.0043) (Table 2).

Regarding S_ne_/S_h_ index it was verified a significant effect of the season and year (Wald χ^2^ = 37.3; df = 6; *p* < 0.001). The index was higher in spring and autumn seasons than in summer (Wald χ^2^ = 23.8 df = 2; *p* < 0.001) and significantly higher in 2018 than in 2017 and 2023 (Wald χ^2^ = 21.1; df = 2; *p* < 0.001). No significant effects of cover crop treatment were detected (Wald χ^2^ = 2.0; df = 2; *p* = 0.360) (Table 2).

Finally, the global model revealed significant effects on the stability index (S_ne_ + S_n_)/S_h_ (Wald χ^2^ = 40.2; df = 6; *p* < 0.001). Nevertheless, neither cover crop treatments (Wald χ^2^ = 1.8; df = 2; *p* = 0.41) nor seasons (Wald χ^2^ = 2.6; df = 2; *p* = 0.27) significantly affected this index. Sampling year was the only significant explanatory variable, with the highest values recorded in 2018 and the lowest in 2023 (Wald χ^2^ = 28.8; df = 2; *p* < 0.001) (Table 2).

### 3.5. Associations Between Cover Crop Vegetation and Soil-Surface Arthropod Communities

Pearson correlation analysis (including all cover crop types, seasons and sampling years) revealed a considerable number of significant correlations between cover crops characteristics and the activity density and diversity of soil surface arthropods communities. Percentage of cover vegetation was positively correlated with arthropod richness, Shannon–Wiener diversity index, Pielou’s evenness index, the effective number of common species (^1^D), the effective number of dominant species (^2^D) and the stability of index Nne/Nh. In contrast, it was negatively correlated with both abundance and Simpson’s diversity index. Plant richness was positively correlated with the Shannon–Wiener diversity index, Pielou’s evenness index, the effective number of common species (^1^D), and the effective number of dominant species (^2^D), whereas it was negatively correlated with Simpson’s diversity index. Although the correlations were statistically significant, their strength was weak (Table 3). As also shown in NMDS analysis this parameter was not correlated with activity density and richness of arthropods.

## 4. Discussion

Cover crops have become an important strategy for promoting biodiversity and enhancing ecosystem services in vineyards [22,31,66,67,68]. Apart from protecting soil against erosion and improving its physical and chemical properties, cover crops provide habitat and food resources for a wide of soil organisms, including arthropods, which have important roles in organic matter decomposition, nutrient cycling, soil structure and pest regulation [66,67,68]. Therefore, understanding how different cover crop types influence soil surface arthropod communities and stability in vineyards is essential for developing more sustainable soil management practices. Most previous studies have focused on activity density, species richness, and some diversity indexes, whereas few have simultaneously evaluated effective species diversity and trophic stability. Such an integrated approach is essential for understanding soil surface arthropod community dynamics and determining whether increases in arthropod activity density translate into more resilient and functional balanced communities, thereby supporting the long-term provision of ecosystem services. It also provides insights into whether the establishment of new cover crop mixtures is necessary or whether the enhancement and sustainable management of spontaneous vegetation can represent a viable alternative, thereby reducing the environmental impacts associated with sowing, particularly soil erosion in steep-slope vineyards.

### 4.1. Plant Community Dynamics Under Different Cover Crop Treatments

The LEG treatment consistently maintained the highest percentage of cover vegetation, while LEGRA showed the lowest species richness throughout the study. Although the percentage of cover vegetation changed significantly among years, species richness remained remarkably stable, indicating that temporal changes in vegetation were driven primarily by shifts in species composition rather than by changes in the number of species present. Seven years after sowing, LEG still provided the greatest soil cover, whereas the lower species richness observed in LEGRA may reflect the progressive dominance of *D. glomerata*. This perennial grass is highly competitive and has also been reported to exhibit allelopathic effects that inhibit the germination and growth of neighboring species, including grasses and legumes [69]. Such competitive interactions may have favored the persistence of *D. glomerata* while reducing the establishment and persistence of other species within the community. In contrast, SPONT maintained plant richness comparable to that of LEG, suggesting that naturally assembled plant communities can sustain levels of species richness similar to those achieved by sown legume mixtures. LEG was the only treatment that consistently exceeded the 30% of coil cover threshold recommended for effective soil protection in Mediterranean vineyards [70], highlighting its potential to reduce soil erosion while providing other ecosystem services, such as improving soil nutrient availability and supporting vine productivity.

The reduction in the number of sown species in LEG and LEGRA treatments, over time, including some initially observed as indicator species, suggests that only a few numbers of species (*T. subterraneum* and *T. michelianum* in both treatments, and *D. glomerata* in LEGRA treatment) were able to persist under the local environmental conditions and regular vineyard management, while the remaining species gradually disappeared from the community. Similar patterns have been reported in other vineyard and Mediterranean agroecosystems, where the composition of sown cover crops changed progressively over time as local environmental conditions, management practices and interspecific competition filter out poorly adapted species [12,22,71]. The decline of most of the sown species over time does not necessarily indicate failure of the cover crop mixture. In multispecies seed mixtures, annual species that establish rapidly are often included to ensure rapid soil cover and facilitate community establishment [72,73]. Despite these compositional changes, the sown treatments appeared to follow distinct floristic trajectories in accordance with the functional identity imposed by the seed mixtures used in each treatment (LEG remaining dominated by Fabaceae species and LEGRA by Fabaceae and Poaceae species). In contrast, the SPONT treatment exhibited a progressive change in species composition, probably reflecting the combined effects of initial soil disturbance (autumn of 2016), impact of climate conditions of the year, and the subsequent natural recolonization from the soil seed bank.

### 4.2. Associations Between Vegetation Characteristics and Soil Surface Arthropod Communities

Our results showed that soil surface arthropod communities were dominated by Collembola across all treatments and sampling years, with the LEGRA treatment supporting the highest Collembola activity density. Previous studies have demonstrated that both quantity and quality of plant biomass strongly influence arthropod activity density and diversity in agroecosystems [74,75,76,77]. In addition, the mulch layer formed after mowing may further enhance habitat quality by providing shelter, buffering fluctuations in soil temperature and moisture, and serving as food resource for detritivore organisms [78,79,80]. These mechanisms are consistent with the predominance of detritivores, particularly Collembola, which accounted for more than two-thirds of all individuals captured in this study, suggesting that cover crops favored decomposer communities. Similar results were reported in vineyards and other perennial crops, where cover crops have been shown to promote detritivore populations [22,75,81]. By feeding primarily on fungi, bacteria and decaying plant material, Collembola contribute to decomposition processes and nutrient cycling, thereby supporting soil fertility [82].

Although herbivores, omnivores, and zoophages represented a considerably smaller proportion of the soil surface arthropod community, they play functional roles in maintaining ecosystem stability. The predominance of detritivores may directly or indirectly benefit higher trophic levels by increasing the availability of prey for generalist predators. In addition, vegetation cover may provide additional refuges and resources for arthropods, potentially contributing to community stability [76,77]. For instance, Collembola represents an important food resource for generalist predators, particularly spiders and predatory beetles, thereby contributing to the stability and functioning of soil food webs [22].

Although the LEGRA treatment showed the highest activity density of soil surface arthropods, this higher number of individuals was not reflected in greater community diversity: Shannon–Wiener diversity and Pielou’s evenness were significantly higher in LEG than in LEGRA, whereas SPONT differed from LEGRA only in the case of Pielou’s evenness index.

These results demonstrate that a greater number of individuals does not necessarily correspond to a more diverse community, since the high activity density recorded in the LEGRA treatment may have been driven by one or a few dominant taxa. This interpretation was further supported by Simpson’s diversity index, which was significantly higher in LEGRA, indicating greater species dominance, whereas the lower values observed in LEG and SPONT were consistent with a more even distribution among species. The higher soil surface arthropod activity density observed in LEGRA may have been associated with the greater aboveground biomass and the more persistent plant residues remaining after mowing (higher C/N). These conditions may have favored detritivorous taxa, particularly Collembola, resulting in the dominance of a few morphospecies rather than a more even distribution of the arthropod community.

The use of Hill numbers, which express traditional diversity indices as effective numbers of species and thereby provide a more intuitive interpretation of diversity [83], confirmed these patterns. The lower values of ^1^D in the LEGRA treatment indicate that, despite its higher activity density, the community was dominated by relatively few common and abundant species. Likewise, the lower value of ^2^D suggests that the community was dominated by a limited number of abundant species, reducing its functional complexity. In contrast, LEG and SPONT supported higher effective numbers of both common and dominant species, indicating a more balanced community composition.

Seasonal and interannual variation exerted a strong influence on soil surface arthropod communities, often exceeding the effects of cover crop type. Spring consistently supported higher activity density, richness and ^1^D than summer and autumn, probably reflecting the favorable combination of moderate temperatures, higher soil moisture and greater vegetation. In contrast, autumn generally exhibited lower activity density, Shannon and ^1^D likely as a consequence of the prolonged summer drought that characterizes the region. Higher Pielou’s evenness in summer and autumn indicated a more even community structure, whereas spring was characterized by greater species dominance, as also reflected by the higher Simpson’s index and ^2^D.

The NMDS ordination showed considerable overlap among the three treatments, indicating that treatment had only a limited effect on soil surface arthropod community composition. In contrast, samples tended to separate according to sampling year, particularly those collected in 2023, suggesting that temporal variation exerted a stronger influence on community composition than cover crop treatment. Nevertheless, the fitted environmental vector indicated that vegetation cover was associated with the observed community patterns, suggesting that vegetation structure contributed to arthropod assemblage composition, although to a lesser extent than temporal variation.

Marked interannual differences were also observed and may have been associated with contrasting climatic conditions. The exceptionally warm and dry conditions recorded in 2017, characterized by prolonged heat waves and prolonged drought conditions [84], may have limited soil surface arthropod activity density despite the relatively high species richness and diversity observed in that year. In contrast, the cooler and exceptionally wet spring of 2018 [85] may have promoted vegetation growth and soil moisture, creating favorable conditions for soil surface arthropods. Although 2023 was also characterized by an extremely hot and dry spring, the month of May was marked by the unusually frequent occurrence of several adverse weather events, particularly periods of heavy rainfall [86]. Nevertheless, soil surface arthropod activity density remained higher than in the other years. This apparent difference may reflect not only climatic conditions during the sampling period, but also differences in antecedent environmental conditions, vegetation development, and community composition between years. The higher activity density recorded in 2023, despite lower richness and diversity, suggests that this pattern may have been driven by increased arthropod activity or dominance of relatively small number of taxa rather than by a more diverse community.

The stability indices provided additional insight into the functionality of soil surface arthropod communities by measuring the balance between herbivorous, zoophagous and neutral arthropods. Overall, the lack of significant differences among cover crop treatments for most stability indices suggests that changes in vegetation composition had only a limited influence on the trophic stability of soil surface arthropod communities. Similar results have been reported previously, suggesting that different cover crop management strategies may alter community composition without substantially altering its functional stability [22].

The absence of significant differences in the N_ne_/N_h_ and Sne/S_h_ indices among treatments may reflect the high degree of functional redundancy within soil surface arthropod communities. These indices are used to evaluate the inhibitory effects of zoophagous on the herbivore’s population [56]. The observed patterns may therefore result from the functional compensation capacity of predator communities, whereby different species play similar ecological roles, maintaining the regulation of herbivores despite changes in species composition [22,87,88]. Such functional redundancy is recognized as an important mechanism contributing to ecosystem resilience by buffering the effects of environmental disturbances and helping to sustain ecosystem functioning and services in managed agroecosystems [89,90].

Only the (Nne + Nn)/Nh index differed significantly among cover crop treatments. By incorporating neutral arthropods (detritivores and omnivores), this index reflects the contribution of these groups to trophic stability through their interactions with both herbivores and zoophagous [56]. The higher values observed in SPONT suggest that this treatment may favor a more balanced and functionally soil surface arthropod community. Neutral arthropods, such as Collembola, may contribute indirectly to ecosystem functioning by sustaining detrital food webs and providing alternatives for generalist predators thereby promoting more stable trophic interactions and enhancing herbivore regulation. A significantly higher value for the (Nne + Nn)/Nh index was reported for the sown cover crop compared with the spontaneous cover crop [22]. Interestingly, all stability indices remained above one regardless of treatment. Values above this threshold indicate a potential regulatory influence of zoophagous arthropods on herbivore populations [56]. This finding suggests that all treatments allowed for relatively balanced trophic conditions within the soil surface arthropod communities.

Trophic stability varied strongly among seasons and sampling years, suggesting that climatic conditions may have contributed to variation in the functional structure of soil surface arthropod communities. Most stability indices were higher in spring and autumn than in summer, indicating that favorable climatic conditions promoted more balanced trophic interactions, whereas summer drought and high temperatures reduced community stability. Interannual differences were evident, with the highest stability recorded in 2018, likely reflecting the favorable cooler and wetter conditions, whereas the warm and dry conditions of 2017 and 2023 probably constrained trophic stability.

The significant positive correlations observed between cover crop characteristics and most diversity metrics further support the role of vegetation in shaping soil surface arthropod communities. Although these relationships were weak, they consistently indicated that increasing plant richness and percentage of soil cover favored more diverse and even arthropod assemblages. Our study suggests that vegetation cover and plant richness may play a more important role in structuring soil surface arthropod communities than the identity of the cover crop treatment itself. These findings agree with previous studies showing that greater vegetation complexity increases habitat heterogeneity, food availability and refuge, thereby supporting a wider range of arthropod taxa [75,76,77].

An important limitation of the present study is the lack of treatment randomization. Because the three treatments were established as large, continuous blocks, spatial heterogeneity in the distribution of soil surface arthropods may have contributed to differences among treatment blocks and cannot be completely separated from treatment effects. This limitation should therefore be considered when interpreting the observed differences among treatments. Future studies using replicated and randomized treatment designs would help to further disentangle treatment effects from spatial variation.

## 5. Conclusions

Overall, the present study demonstrates that cover crop types were associated with differences in the activity density, diversity, and trophic structure of soil surface arthropod communities in Mediterranean vineyards. Although LEGRA showed the highest soil surface arthropod activity density, both Shannon–Wiener diversity and Pielou’s evenness were higher in LEG than in LEGRA. For Shannon–Wiener diversity, no significant difference was detected between LEGRA and SPONT, whereas for Pielou’s evenness, LEGRA differed significantly from both LEG and SPONT. Trophic stability indices indicated a relatively balanced trophic structure across all cover crop treatments. These findings highlight that activity density alone provides only a partial overview of community responses and reinforce the importance of integrating complementary biodiversity metrics to obtain a more comprehensive assessment of soil surface arthropod communities.

Our findings highlight the ecological value of spontaneous vegetation, suggesting that it should be regarded as an important component of vineyard management capable of supporting diverse and functionally balanced soil surface arthropod communities.

While sown cover crop mixtures may facilitate rapid vegetation establishment and improve soil cover under adverse environmental conditions, conserving well-managed spontaneous vegetation represents a low-input and valuable alternative for Mediterranean vineyards. Because spontaneous plant communities are naturally more efficient to local climatic and edaphic conditions, they generally require less intervention and may contribute to maintaining resilient agroecosystems and long-term ecosystem functioning [22,91].

Future studies should adopt a more holistic approach by simultaneously assessing multiple ecosystem services, including nutrient cycling, soil physical and biological properties, pest regulation and grapevine productivity, to identify management strategies that maximize ecosystem multifunctionality without compromising yield. Recent evidence indicates that well-managed cover crops can enhance regulating and supporting ecosystem services while maintaining grape production, highlighting their potential as a key component of sustainable viticulture [12,92].

## Figures and Tables

**Table 1:** Results of the generalized linear mixed models evaluating the effect of cover crop treatment, season and sampling year on the soil surface arthropod variables (AD—activity-density; S—richness; H’—Shannon–Wiener diversity index; SDI—Simpson’s diversity index; J—Pielou’s evenness index; 1D—effective number of common species; 2D—effective number of dominant species) analyzed. Mean (SE) of each parameter are shown for each treatment. Means followed by different letters are significantly different at *p* < 0.05. * Indicates significant differences for *p* < 0.05.

	Global Wald χ^2^	df	*p* Value	Cover Crop Treatment			Season	Year
				LEGRA	SPONT	LEG		
AD	47,655.4	6	*p* < 0.001	389 (85.86) ^a^	169.7 (25.44) ^b^	198.61 (45.45) ^b^	*	*
S	361.8	6	*p* < 0.001	17.26 (0.65) ^a^	16.85 (0.58) ^a^	17.88 (0.62) ^a^	*	*
H’	43.8	6	*p* < 0.001	1.64 (0.06) ^b^	1.85 (0.06) ^ab^	1.99 (0.05) ^a^	*	*
SDI	32.5	6	*p* < 0.001	0.36 (0.02) ^a^	0.29 (0.02) ^b^	0.25 (0.02) ^b^	*	*
J	46.3	6	*p* < 0.001	0.60 (0.02) ^b^	0.67 (0.02) ^a^	0.71 (0.02) ^a^	*	*
^1^D	75.1	6	*p* < 0.001	6.42 (0.38) ^b^	7.41 (0.35) ^ab^	8.52 (0.41) ^a^	*	*
^2^D	41.7	6	*p* < 0.001	4.26 (0.27) ^b^	4.99 (0.26) ^ab^	5.78 (0.31) ^a^	*	*

**Table 2:** Results of the generalized linear mixed models evaluating the effects of cover crop type, season and sampling year on the stability indices of the soil surface arthropods community. Mean (SE) of each parameter are shown for each treatment. Means followed by different letters are significantly different at *p* < 0.05. * Indicates significant differences for *p* < 0.05, while n.s. indicates non-significant differences.

	Global Wald χ^2^	d.f	*p* Value	Cover Type			Season	Year
				LEGRA	SPONT	LEG		
N_ne_/N_h_	39.7	6	*p* < 0.001	1.41 (0.23) ^a^	1.74 (0.19) ^a^	1.76 (0.28) ^a^	*	*
(N_ne_ + N_n_)/N_h_	20.1	6	*p* = 0.003	25.33 (4.74) ^ab^	30.72 (7.86) ^a^	11.52 (2.12) ^b^	*	*
S_ne_/S_h_	37.3	6	*p* < 0.001	2.19 (0.12) ^a^	2.40 (0.15) ^a^	2.19 (0.14) ^a^	*	*
(S_ne_ + _Sn_)/S_h_	40.2	6	*p* < 0.001	5.23 (0.23) ^a^	5.58 (0.30) ^a^	5.16 (0.27) ^a^	n.s.	*

**Table 3:** Pearson’s correlation analysis between percentage of cover vegetation and plant richness (all cover crops join) and soil surface arthropods variables. Significant correlations are indicated for *p* < 0.05, while n.s. indicates non-significant differences.

	Cover Vegetation (%)		Plant Richness	
	r	*p*	r	*p*
Richness	0.110	0.035	−0.006	n.s.
Activity-density	−0.126	0.016	−0.055	n.s.
H’	0.186	<0.001	0.131	0.012
SDI	−0.128	0.015	−0.130	0.013
J	0.158	0.002	0.141	0.007
^1^D	0.246	<0.001	0.111	0.034
^2^D	0.231	<0.001	0.104	0.046
N_ne_/N_h_	0.106	0.049	0.097	n.s.
(N_ne_ + N_n_)/N_h_	−0.069	n.s.	0.000	n.s.
S_ne_/S_h_	0.034	n.s.	0.046	n.s.
(S_ne_ + S_n_)/S_h_	0.071	n.s.	0.061	n.s.

## Data Availability

The data presented in this study are available from the corresponding author upon reasonable request.

## Supplementary Materials

The following supporting information can be downloaded at: https://www.mdpi.com/article/10.3390/insects17090975/s1, Figure S1: Location of the vineyard where the study was conducted, and experimental design showing the location of the vegetation surveys and pitfall, indicated by circles. LEGRA—sown with a commercial mixture of Fabaceae and Poaceae; SPONT—spontaneous vegetation was allowed to develop naturally; LEG—sown with a commercial mixture of Fabaceae; Figure S2: Relative abundance (%) of the different functional and taxonomic groups captured, according to treatment (a) and survey year (b). D—detritivore; H—herbivore; NE—natural enemy; O—omnivore; Ind—indeterminate; Aran—Araneae; Coll—Collembola; Col—Coleoptera; Hemi—Hemiptera; Hym—Hymenoptera; Isop—Isopoda; Ort—Orthoptera; Opil—Opiliones; Orib—Oribatida; Table S1: Plant species recorded during the floristic surveys conducted in 2017, 2018 and 2023 in each cover crop treatment. Values indicate the number of sampling points (out of 10) where each species was recorded. Sown species are shown in bold; Table S2: Significant indicator species and their IndVal scores for the vegetation treatments LEGRA, LEG and SPONT across the three sampling years. Abbreviations are defined in Supplementary Table S1; Table S3. Arthropod species richness and abundance across taxonomic and trophic groups; Table S4. Mean ± standard error (SE) of the abundance and richness of the different trophic groups per pitfall trap in each sampling year and treatment.
