# Supplementary material for: Vegetation and Soil Surface Arthropods in Mediterranean Vineyards with Different Interrow Cover Crop Combinations
Source: Insects. 2026 Sep 21;17(9):975. doi: 10.3390/insects17090975 (PMC13607022; doi:10.3390/insects17090975)
Supplement: Supplementary file 1 [file insects-17-00975-s001.zip › insects-4506032-supplementary.pdf]

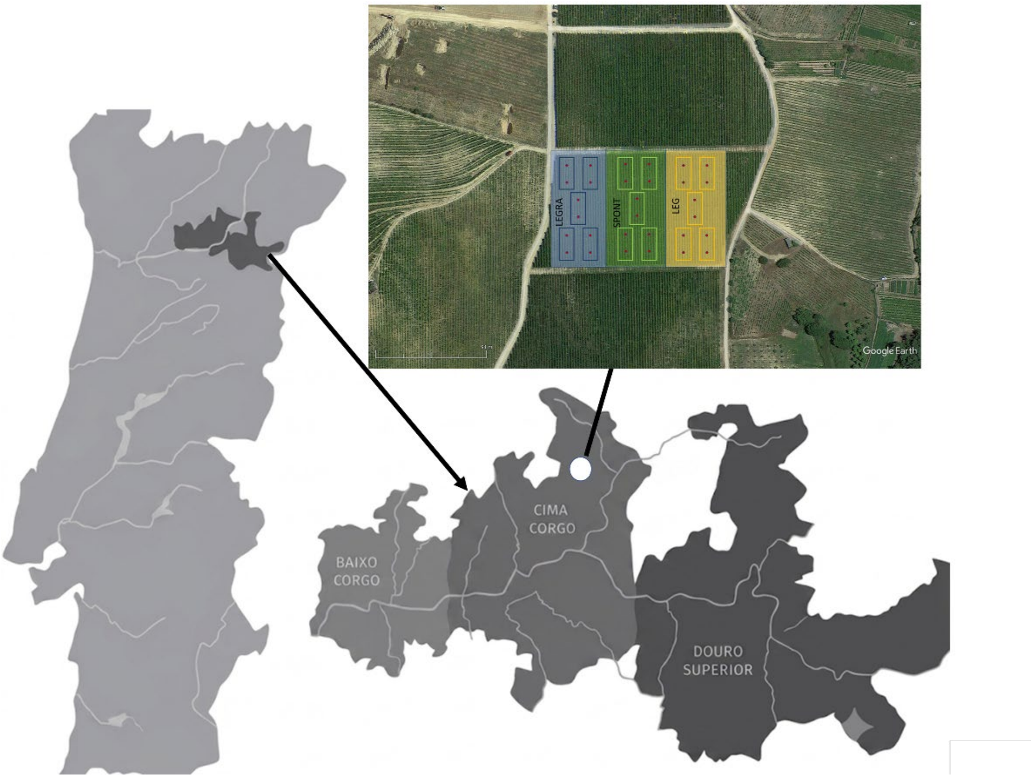

Figure S1. Location of the vineyard where the study was conducted, and experimental design showing the location of the vegetation surveys and pitfall, indicated by circles. LEGRA - sown with a commercial mixture of Fabaceae and Poaceae; SPONT - spontaneous vegetation was allowed to develop naturally; LEG - sown with a commercial mixture of Fabaceae.

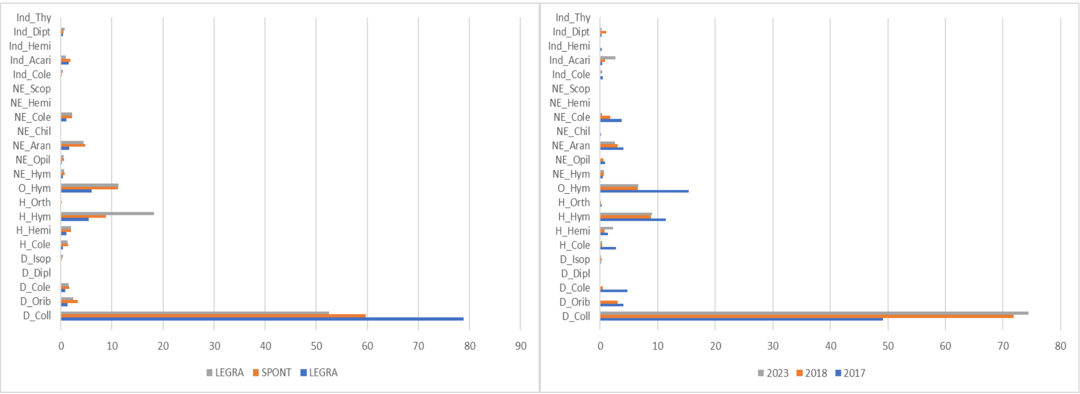

(a) (b)

Figure S2. Relative abundance (%) of the different functional and taxonomic groups captured, according to treatment (a) and survey year (b). D – detritivore; H – herbivore; NE – natural enemy; O – omnivore; Ind - Inderterminated; Aran – Araneae; Coll – Collembola; Col – Coleoptera; Hemi – Hemiptera; Hym – Hymenoptera; Isop – Isopoda; Ort - Orthoptera; Opil – Opiliones; Orib - Oribatida

Table S1. Plant species recorded during the floristic surveys conducted in 2017, 2018 and 2023 in each cover crop treatment. Values indicate the number of sampling points (out of 10) where each species was recorded. Sown species are shown in bold.

Code	Species	Cover crop/ Year								
		LEGRA			LEG			SPONT		
		2017	2018	2023	2017	2018	2023	2017	2018	2023
Ana.arv	*Anagallis arvensis* L.							2	3	1
And.int	*Andryala integrifolia* L.		6	7	3	2	4	3	1	7
Ant.arv	*Anthemis arvensis* L.	2		1	9		8	7		3
Alc.mic	*Alchemilla microcarpa* Boiss. & Reut.				1					2
Ara.tha	*Arabidopsis thaliana* (L.) Heynh.	1			1					1
Ave.bar	*Avena barbata* Pott ex Link				1	3	2			
Bra.bar	*Brassica barrelieri* (L.) Janka	4	2		10	9	5	8	8	2
Bro.dia	*Bromus diandrus* Roth.				1		4			2
Bro.tec	*Bromus tectorum* L.			1						
Cap.bur	*Capsella bursa-pastoris* (L.) Medik.			1				1		
Car.hir	*Cardamine hirsute* L.			1						
Cer.glo	*Cerastium glomeratum* Thuill.	10	1	4	10		3	10		5
Cla.mix	*Cladanthus mixtus* (L.) Chevall.	5	6			8			8	
Cho.jun	*Chondrilla juncea* L.	2	3	1		1	3	2		1
Col.myc	*Coleostephus myconis (L.)* Rchb.f.	1	1	1	2		5	2	4	5
Con.arv	*Convolvulus arvensis* L.	1	2	4	2	2	6	7	6	5
Eri.sum	*Erigeron sumatrensis* Retz.	2	1	9	1		10	7	4	9
Cre.cap	*Crepis capillaris* (L.) Wallr.	1	5	5	5	2	6		5	4
Cyn.dac	*Cynodon dactylon* (L.) Pers.		2	2	1	4	8	2	2	8

**Dac.glo**	***Dactylis glomerata*** L.	1	5	10		1		3	3	1
Ero.cic	*Erodium cicutarium* (L.) L'Hér.		1	2	1		2		2	4
Fes.rub	*Festuca rubra* L.			1						
Gal.par	*Galium parisiense* L.			10	4	4	10	3	7	10
Gam.cal	*Gamochaeta calviceps* (Fernald) Cabrera			2			1			
Ger.dis	*Geranium dissectum* L.						4	1		9
Ger.mol	*Geranium molle* L.			2	2	5	4			
Ger.pur	*Geranium purpureum* Vill.					1			3	
Hol.lan	*Holcus lanatus* L.				1					
Hor.mur	*Hordeum murinum* subsp. *leporium* (Link) Arcang.		1				1			
Hyp.per	*Hypericum perforatum* L.	1				1	2			
Hyp.rad	*Hypochaeris radicata* L.		5	6	1	1	4	7	1	7
Jas.mon	*Jasione montana* L.	1								
Jun.buf	*Juncus bufonis* L.			2						1
Lam.amp	*Lamium amplexicaule* L.				1			4		
Lat.ang	*Lathyrus angulatus* L.							1	1	
Leo.rot	*Leontodon rothii* Ball						1			
Leo.sax	*Leontodon saxatilis* Lam. subsp. *saxatilis*		2						1	
**Lol.mul**	***Lolium multiflorum*** Lam.	9	10			3		2		
Lol.rig	*Lolium rigidum* Gaudin		1	4	5		3	3		2
Med.pol	*Medicago polymorpha* L.		1	8						
Mol.min	*Molineriella minuta* (L.) Rouy	5	3		1	1		4	1	4
Mus.com	*Muscari comosum* (L.) Mill.					1				1
Mys.arv	*Myosotis arvensis* (L.) Hill				1					

Orn.com	*Ornithopus compressus* L.	1	8	3	3	8	9		7	3
**Orn.sat**	***Ornithopus sativus*** Brot.	8	7		7	9		1	6	
Pha.lan	*Phacelia tanacetifolia* Benth.				1					
Pla.cor	*Plantago coronopus* L.			3		1	3	3	1	5
Pla.lan	*Plantago lanceolatus* L.			2			5	1	2	7
Poa.ann	*Poa annua* L.	3		3	3	3		5	7	2
Pol.avi	*Polygonum aviculare* L.				1			1		
Ran.arv	*Ranunculus arvensis* L.							2		
Rap.rap	*Raphanus raphanistrum* L.	3	3		5	1	2	2		
Res.lut	*Reseda luteola* L.					2				
Rum.ace	*Rumex acetosella* subsp. *angiocarpus* (Murb.) Murb	5	6	4	8	9	5	8	10	3
Scl.ann	*Scleranthus annus* L.				3	4		1	4	
Sen.vul	*Senecio vulgaris* L.				2	1		6		
Sil.gal	*Silene gallica* L.	8	5	7	2	4	6	7	8	3
Sol.nig	*Solanum nigrum* L.		2			1			1	
Son.asp	*Sonchus asper* L.			1						
Spe.arv	*Spergula arvensis* L.	8	3	1	9	6		7	4	
Ste.med	*Stellaria media* (L.) Vill.					1				
Tri.cam	*Trifolium campestre* Screb.					1			1	
Tri.glo	*Trifolium glomeratum* L.			1						
**Tri.inc**	***Trifolium incarnatum*** L.	8	5		9	9			3	
**Tri.ist**	***Trifolium isthmocarpus*** Brot.	6			9			2	1	
**Tri.mic**	***Trifolium michelianum*** Savi	4	4	1		8	1		5	
Tri.rep	*Trifolium repens* L.	1			1					

**Tri.res**	***Trifolium resupinatum*** L.	9	9		9	10		1	7	
**Tri.sub**	***Trifolium subterraneum*** L.	3	9	1	3	9	10	7	10	3
Tri.ves	*Trifolium vesiculosum* Savi								2	
Ver.arv	*Veronica arvensis* L.		1	1	3	2	2	6	3	3
Vio.arv	*Viola arvensis* Murray			1			1			
Vio.kit	*Viola kitaibeliana* Schult.				3	1		2	2	
Vul.bro	*Vulpia bromoides* L.		9	9	3	10	10	8	10	10

Table S2. Significant indicator species and their IndVal scores for the vegetation treatments LEGRA, LEG and SPONT across the three sampling years. Abbreviations are defined in Supplementary Table S1.

Indicator	LEGRA			SPONT			LEG			2017-2023		
	2017	2018	2023	2017	2018	2023	2017	2018	2023	LEGRA	SPONT	LEG
**Lol. mul**	**0.818*** **	**0.793 *** **								**0.553*** **		
Cla. mix	0.500**							0.527*				
**Tri. mic**	**0.400***											
**Dac. glo**			**0.976*** **							**0.456*** **		
Med. pol			0.800***							0.300***		
Hyp. rad				0. 656***							0.291*	
Vul. bro				0.605**							0.392*	
**Tri. sub**				**0.586***					**0.918*** **			**0.351***
Con. arv				0.539**							0.326**	
Eri. sum				0.450*	0.375*							
Sen. vul				0.446*								
Ger. dis						0.656***					0.347**	
Mol. min						0.400*						
Poa ann					0.459*						0.269*	
Gal. par					0.417*						0.317*	
Ver. arv											0.222*	
**Tri. ist.**							**0.628** **					**0.205***
**Tri. inc**							**0.600** **	**0.578** **				**0.397*** **
**Tri. mic**								**0.527***				
Ant. arv							0.500*		0.505*		0.200**	0.404**
**Orn. sat**							**0.495***					**0.306***
Orn. com									0.605**			0.350**
Cre. cap							0.417*					
Bra. bar								0.531*				0.417***
**Tri. res**								**0.508** **				**0.335***
Ger. mol								0.500**				0.316***

Table S3. Arthropod species richness and abundance across taxonomic and trophic groups.

Trophic group	Taxonomic level<br>Class	Order/ Suborder	Superfamily/Family	Nº individuals	Nº species/<br>morphospecies
Detritivorous	Arachnida	Acari/ Oribatida		1873	1
	Diplopoda			13	1
	Entognatha	Collembola		62935	4
	Insecta	Coleoptera	Aderidae	31	1
			Anthicidae	917	6
			Corylophidae	3	1
			Latridiidae	10	1
			Scarabaeidae	43	1
			Tenebrionidae	158	3
		Blattodea		1	1
	Isopoda			158	2
Herbivorous	Insecta	Coleoptera	Carabidae	75	3
			Chrysomelidae	226	5
			Curculionidae	153	17
			Dasytidae	1	1
			Elateridae	361	1
			Nitidulidae	1	1
			Phalacridae	5	1
		Hemiptera	Aphididae	511	1
			Cercopidae	7	1
			Cicadellidae	650	2
			Cydnidae	169	3
			Psyllidae	3	1
			Lygaeidae	53	1
			Pentatomidae	4	1
		Hymenoptera	Formicidae	8801	1
		Orthoptera	Acrididae	29	2
			Gryllidae	78	1
			Gryllotalpidae	17	1
Omnívorous		Hymenoptera	Formicidae	7905	35
Zoophagous	Arachnida	Araneae		2825	173
		Opiliones		362	3
		Scorpiones		1	1
	Chilopoda.			79	1
	Insecta	Coleoptera	Carabidae	1307	46
			Coccinellidae	24	3
			Malachiidae	9	1
			Meloidae	1	1
			Staphylinidae	175	19
		Diptera	Syrphidae	3	1
		Hemiptera	Anthocoridae	2	1
			Nabidae	12	1
		Hymenoptera	Chalcidoidea	253	2
			Chrysididae	2	1
			Dryinidae	4	1
			Icheneumonoidea	3	1
			Mutillidae	57	1
			Pompilidae	11	1
			Vespidae	2	1
			Other Hymenoptera	205	2
Indeterminate	Arachnida	Acari		1363	2
	Insecta	Coleoptera		218	8
		Diptera		496	6
		Hemiptera	Miridae	1	1
			Other Heteroptera	61	1
		Thysanoptera		72	1
**Total**				**92739**	**381**
**Detritivorous**				**66142**	**22**
**Herbivorous**				**11144**	**44**
**Omnívorous**				**7905**	**35**
**Zoophagous**				**5337**	**261**
**Indeterminate**				**2211**	**19**

Table S4. Mean ± standard error (SE) of the abundance and richness of the different trophic groups per pitfall trap in each sampling year and treatment.

Year	Treatment	Herbivorous	Zoophagous	Omnívorous	Detritivorous
			Abundance		
2017	LEGRA	18.48 ± 4.72	11.96 ± 1.64	23.16 ± 2.59	107.36 ± 22.50
	SPONT	22.58 ± 7.78	13.02 ± 1.68	21.78 ± 3.13	90.47 ± 19.88
	LEG	28.45 ± 8.72	15.57 ± 2.25	22.21 ± 3.33	55.51 ± 26.16
2018	LEGRA	16.02 ± 2.92	14.40 ± 1.83	20.00 ± 2.32	181.09 ± 53.16
	SPONT	21.96 ± 5.59	15.16 ± 1.72	14.56 ± 1.84	166.80 ± 61.80
	LEG	38.91 ± 11.35	15.80 ± 2.30	14.68 ± 3.09	228.34 ± 115.20
2023	LEGRA	60.17 ± 20.77	15.87 ± 4.27	29.73 ± 3.24	861.77 ± 324.24
	SPONT	18.07 ± 2.26	15.79 ± 2.51	21.34 ± 3.98	53.90 ± 15.64
	LEG	70.57 ± 25.89	15.27 ± 2.42	33.63 ± 4.36	32.53 ± 10.00
			Richness		
2017	LEGRA	3.30 ± 0.31	6.78 ± 0.67	4.00 ± 0.20	3.56 ± 0.22
	SPONT	3.20 ± 0.30	6.98 ± 0.69	4.07 ± 0.26	3.42 ± 0.26
	LEG	3.77 ± 0.29	7.45 ± 0.73	3.98 ± 0.24	3.15 ± 0.30
2018	LEGRA	2.38 ± 0.26	5.78 ± 0.43	3.29 ± 0.24	3.11 ± 0.18
	SPONT	2.22 ± 0.23	5.29 ± 0.37	3.00 ± 0.23	3.47 ± 0.18
	LEG	2.57 ± 0.23	5.02 ± 0.34	3.11 ± 0.24	3.30 ± 0.19
2023	LEGRA	4.07 ± 0.23	5.93 ± 0.65	3.27 ± 0.16	1.67 ± 0.18
	SPONT	3.79 ± 0.24	6.41 ± 0.47	2.93 ± 0.19	1.48 ± 0.11
	LEG	4.27 ± 0.22	6.83 ± 0.61	3.37 ± 0.21	1.67 ± 0.16
